# Factors influencing online orthopedic doctor–patient consultations

**DOI:** 10.1186/s12911-021-01709-1

**Published:** 2021-12-13

**Authors:** Ping Lei, Jianjun Zheng, Yun Li, Zhongjiang Li, Fei Gao, Xuesong Li

**Affiliations:** Department of Orthopedics, Zhijiang Hospital of Traditional Chinese Medicine, Zhijiang, 443200 Hubei Province China

**Keywords:** Online doctor–patient consultation, Orthopedic consultation, Online medical community, Intention to consult, Perceived value, Perceived trust

## Abstract

**Background:**

Online doctor–patient consultation is a new option for orthopedic patients in China to obtain a diagnosis and treatment advice. This study explores the factors associated with online consultation to formulate operational guidelines for managing online consultations in an online medical community (OMC).

**Methods:**

An empirical model was developed to identify the factors that influence online orthopedic doctor–patient consultations in an OMC while focusing on the perceived value of and perceived trust in online consultations. The moderating effects of different risk categories of orthopedic diseases were also considered. Data from 339 feedback surveys from orthopedic patients who used online consultation services and Stata software version 14.0 were used to estimate the model parameters and test the robustness of the empirical model.

**Results:**

Of those who completed the feedback surveys, 53.42% were female patients, 82.27% were between 18 and 60 years old, and 61.98% sought consultations online more than 2 times per year. Model analysis demonstrated that the regression coefficients of the perceived value of and perceived trust in online consultations are 0.489 (*p* < 0.01) and 0.505 (*p* < 0.01), respectively. The interaction coefficient between disease risk and perceived value is 0.336 (*p* < 0.01), and the interaction coefficient between disease risk and perceived trust is − 0.389 (*p* < 0.01).

**Conclusions:**

Orthopedic patients’ perceived value of and perceived trust in online consultations in an OMC can significantly influence their intention to seek online disease diagnosis and treatment consultations. The effects of perceived value and perceived trust on patients' intention to consult vary significantly across different disease risk categories. Therefore, enhancing the perceived value and perceived trust of orthopedic patients is an important component of OMC operation and management.

**Supplementary Information:**

The online version contains supplementary material available at 10.1186/s12911-021-01709-1.

## Background

Electronic consultation services are growing rapidly worldwide. Consultation methods include telephone consultations, online video, and real-time or non-real-time informational interactions in online medical communities. An online medical community (OMC) refers to doctor–patient communications centered on medical information services using information and communication technology (ICT) [1, 2]. Patients can send texts, pictures, videos and other information to doctors for consultation, and then, doctors diagnose the patient's condition and provide relevant suggestions. A growing number of orthopedic patients in China are consulting doctors through an OMC when seeking medical treatment. In 2019, there were more than 30 million consultations on Good Doctor Online (https://www.haodf.com), the online medical community with the largest number of users in China [3].

Related studies have explored the use of electronic consultation in orthopedic practice and the acceptance and satisfaction of patients and doctors with electronic consultations, a nonphysical contact information exchange method. Foni et al. proposed that there are multiple appropriate electronic consultation services available for orthopedic consultations [4], such as teleconsultation, for diagnosis, treatment, patient follow-up, or virtual rehabilitation; orthopedists can even offer specialized advice to nonspecialist doctors via teleconsultation. On the one hand, electronic consultations provide an opportunity for orthopedic patients to seek medical advice. The use of electronic consultations has advantages such as low costs and avoids unnecessary traveling, parking and waiting [5, 6], and it relieves pressure on the medical system [7]. Buvik et al. showed that it is cost-effective from social and health sector perspectives to provide video-assisted orthopedic consultations to remote clinics [8] rather than having patients travel to specialist hospitals for consultations. On the other hand, electronic consultation plays a role in orthopedic patients obtaining relevant knowledge before visiting a hospital. Thomas et al. previously demonstrated the importance of electronic consultation as a tool for preoperative education in arthroplasty patients [9]. Claassen et al. also proposed that use of an electronic consultation tool to prepare for the first orthopedic consultation for hip or knee osteoarthritis influenced cognition about osteoarthritis [10]. In addition, electronic consultation has become an important way to provide nonemergency orthopedic consultation during special periods [11]. Mignela et al. pointed out that, during the coronavirus disease 2019 (COVID-19) pandemic, electronic consultation could be further promoted and used, reducing the risk of infection and ensuring the safety of orthopedic surgeons and patients [12].

The use of various applications, such as WhatsApp and MyDoc, has been accepted by an increasing number of doctors and patients [13, 14]. As a popular electronic consultation tool, the OMC is favored by doctors and patients due to its convenience, ease of use, and low cost. However, both industry applications and theoretical research have lacked in-depth discussions of how to improve OMC information consulting services to increase their use.

This study explores the factors associated with patient intention to seek orthopedic consultations in the OMC from two main aspects: patient perceived value and perceived trust. Keith et al. believed that orthopedic patients’ perceived value of technology will affect their acceptance of such technology [15]. Orthopedic patients exchange information with orthopedic surgeons via the OMC tool, hoping to obtain valuable information. Patients' perception of the value of information has a significant direct impact on their intention to consult in an OMC [16]. Therefore, we posit the following hypothesis:

**H1**: The greater the perceived value of an OMC is, the greater orthopedic patients’ intention to seek consultations in an OMC.

Due to people’s demand for high accuracy, privacy and sensitivity of medical and health information, trust is particularly important in the field of medical and health services. Trust is necessary for building doctor–patient relationships [17]. Audrain-Pontevia and Menvielle found that trust is an important factor affecting the use of an OMC [18]. Hence, we posit the following hypothesis:

**H2**: The greater the perceived trust in an OMC is, the greater orthopedic patients’ intention to seek consultations in an OMC.

Notably, even for nonurgent orthopedic diseases, orthopedic diseases of different risk categories have different effects on patients' intention to use OMC for consultation. For example, the perceived trust in an OMC of patients with lumbar muscle strain could have a greater impact on their intention to seek consultation than that of patients with pelvic fractures. Therefore, in this study, the risk category of orthopedic diseases was used as a moderator to explore the moderating effect of the risk categories on the perceived value and perceived trust of patients and their intention to consult in an OMC. We posit the following hypotheses:

**H3**: The disease risk category has a moderating effect on the impact of perceived value on the patients’ intention to consult.

**H4**: The disease risk category has a moderating effect on the impact of perceived trust on the patients’ intention to consult.

In addition, an OMC often displays relevant information regarding the online doctors, such as their professional title, the number of consultations, and their favorability rating, providing a reference for users when choosing online doctors. According to related research, this type of relevant information can affect users’ behavior [19]. Therefore, to eliminate these interference factors, variables, such as the professional title of doctors, the number of consultations, and the favorability rating of doctors in the OMC, were used as control variables.

## Methods

### Model and variable measurement

**Fig. 1:**
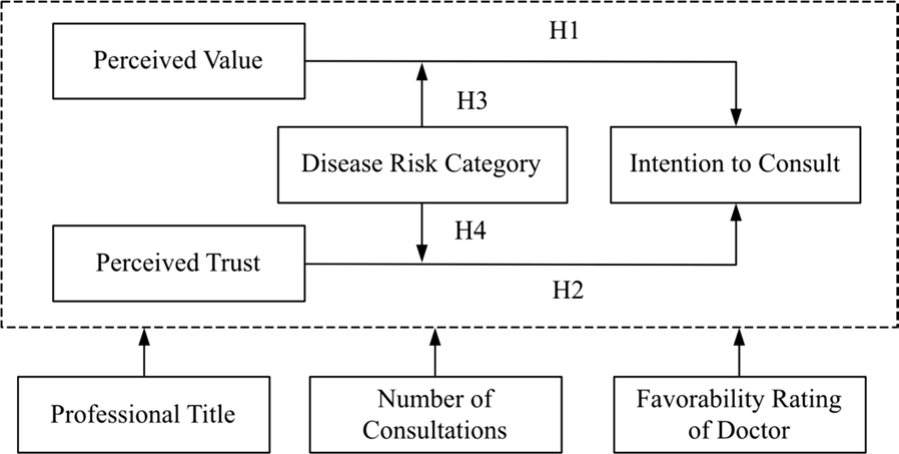
The model of factors influencing orthopedic patients’ intention to consult in an OMC

To explore the impact of perceived value and perceived trust on orthopedic patients’ intention to seek consultations in an OMC, we proposed the model presented below in Fig. 1, which shows the relationship between the variables.

The influence of perceived trust and perceived value on intention has been studied in different field. We drew on the measurement methods for perceived trust, perceived value, and intention to consult developed in related studies. Intention to consult is measured by continuing to consult and recommending others to consult [20]. Based on the relevant research concerning perceived value and the characteristics of doctor–patient interactions in an OMC, perceived value is evaluated based on emotional value, social value, and functional value [21, 22]. Perceived trust is mainly measured based on trust in online doctors and trust in the platform [23]. Disease risk categories are mainly divided into high-risk disease and low-risk disease. The categories of disease risk are marked in the questionnaire to provide a reference for the participants who filled out the questionnaire. The control variables of this study included the professional title of the doctor, the number of consultations and the favorability rating of the doctor in the OMC. The questions regarding the control variables were completed by the participants based on their consultation in the OMC.

The dependent variable in this study is the intention to consult. The independent variables in this study are perceived value and perceived trust. This study uses orthopedic disease risk as a moderator variable. Table 1 lists the descriptions of the variables. They were measured by different dimensions according to the relevant literature and existing instruments or frameworks. In addition, the items were developed by the researchers. Each item was measured on a 5-point Likert scale (1 = strongly disagree; 2 = disagree; 3 = neither agree nor disagree; 4 = agree; 5 = strongly agree).

**Table 1:** Variable descriptions

Variable	Measurement	Item	Source
Intention to consult (IC)	Continue to consult	“*If I need an orthopedic treatment in the future, I will continue to use the consultation service in the online medical community*”	[20]
	Recommend others to consult	*“I would recommend that other users who need orthopedic treatment seek a consultation in the online medical community”*	
Perceived value (PV)	Functional value	*“The online medical community can provide professional and reliable online orthopedic consulting services, and the price is reasonable”*	[21, 22]
	Emotional value	*“The orthopedic consultation service provided by the online medical community is satisfying. The consultation made me feel relaxed and was enjoyable”*	
Perceived trust (PT)	Trust in doctors	*“The doctors in the online medical community will do their best to solve my problems and keep my consultation information confidential.”*	[23]
	Trust in platform	*“The online medical community will try its best to solve the problems during my consultation and keep my personal information confidential”*	
Disease risk category (risk)	High risk	Fracture of cervical vertebrae, pelvic fracture, deep venous thrombosis of lower limb, open fracture, dismemberment of limbs and trunk, etc	[24]
	Low risk	Fasciitis, lumbar muscle strain, heel pain, tenosynovitis, soft tissue injury, external humeral epicondylitis, etc	
Professional title (Title)	Four categories	Resident, attending physician, associate chief physician, chief physician	[25]
Number of consultations (number)	Four levels	100 or below, 100–300, 300–500, 500 or above	[24]
Favorability rating of doctor (rate)	Four levels	70% or below, 70–80%, 80–90%, 90% or above	[26]

As moderator variables, orthopedic diseases were defined as high-risk and low-risk diseases. This research used dummy variables to indicate the categories of disease risk; high-risk disease was expressed as 1, and low-risk disease was expressed as 0. In an OMC, doctors’ professional titles are divided into resident, attending physician, associate chief physician and chief physician. These titles are expressed as 1, 2, 3, and 4, respectively. The number of consultations was divided into four levels, 100 or below, 100–300, 300–500, and 500 or above, which were expressed as 1, 2, 3, and 4, respectively. The favorability rating of the doctor was divided into four levels, 70% or below, 70–80%, 80–90%, and 90% or above, which were expressed as 1, 2, 3, and 4, respectively.

### Study design

To explore the factors influencing orthopedic patients to consult in an OMC, we designed a questionnaire for investigation and analysis. The questionnaire consisted of three parts: an introduction to this study, the basic information of the participants, and the participants' OMC consultations. The introduction to this study mainly informed participants about the nature of the study, the study aims and the confidentiality policy. The basic information included the participants’ gender, age, level of education, and frequency of consultation in the OMC. The questions about participants' OMC consultation in the questionnaire included questions about the specific diseases requiring consultation, perceived value, perceived trust and intention to seek a consultation. We selected 30 orthopedic patients who were in the hospital as presurvey participants to test the quality of the questionnaire. Based on the presurvey results, we adjusted the questionnaire’s content and structure. The final questionnaire and a detailed list of questions can be found in Additional file 1. Composite reliability (CR) and internal consistency were used as the indicators of questionnaire reliability. Content validity and construct validity were used as the indicators of questionnaire validity.

The survey was carried out in accordance with relevant guidelines and regulations. All experimental protocols were approved by the Zhejiang Hospital of Traditional Chinese Medicine. To collect the questionnaire data, we invited participants who sought orthopedic consultations in an OMC to fill out the questionnaire. Participation in the survey was voluntary and completely anonymous. The questionnaire was collected from January to March 2021 mainly by inviting orthopedic patients who visited Zhijiang Hospital of Traditional Chinese Medicine. Participants 18 years of age or older were invited to participate in the questionnaire survey. When receiving a consultation service, patients perceive the value of the consultation service and the level of trust that they have in the platform and in doctors. Therefore, we invited participants to conduct questionnaire interviews based on their online consultation experience. Before the survey process could advance, we presented the content of this study and required the respondents to confirm their informed consent for participating further in this study.

### Model estimation

This study used Stata software, version 14.0 (StataCorp LLC, College Station, TX, USA), to conduct regression analysis of the influencing factors associated with patient intention to seek an orthopedic consultation in the OMC. We proposed the following empirical model to analyze the influences of perceived value and perceived trust on the intention of orthopedic patients to consult and considered the moderating effect of different categories of orthopedic disease risk:

$${IC}_{i}={\beta }_{0}+{\beta }_{1}{PV}_{\mathrm{i}}+{\beta }_{2}{PT}_{\mathrm{i}}+{\beta }_{3}{Title}_{\mathrm{i}}+{\beta }_{4}{Number}_{\mathrm{i}}+{\beta }_{5}{R\mathrm{ate}}_{\mathrm{i}}+{\beta }_{6}{R\mathrm{isk}}_{\mathrm{i}}+{\beta }_{7}{R\mathrm{isk}}_{\mathrm{i}}*{PV}_{\mathrm{i}}+{\beta }_{8}{R\mathrm{isk}}_{\mathrm{i}}*{PT}_{\mathrm{i}}+{\mu }_{i}$$

where i represents the participant (i = 1, 2, 3……N), ${\beta }_{1}$ to ${\beta }_{8}$ are the parameters to be estimated, and ${\mu }_{i}$ is the error term associated with observation i. The variables ${R\mathrm{isk}}_{\mathrm{i}}*{PV}_{\mathrm{i}}$ and ${R\mathrm{isk}}_{\mathrm{i}}*{PT}_{\mathrm{i}}$ are interaction terms.

## Results

### Demographic characteristics

A total of 365 questionnaires were retrieved, of which 339 were valid, and the recovery rate was 92.88%. Among the patients participating in the survey, there were more female patients than male patients (female = 53.42%, male = 46.58%). Regarding the age structure, patients aged 18–30 years accounted for 41.62%, those aged 31–60 years accounted for 40.65%, and those aged over 60 years accounted for 17.73%. In orthopedic clinical practice, the proportion of elderly patients over 60 is larger than that in other clinical practices; however, the subjects of this study are patients who have online consultation experience. We found that the proportion of orthopedic patients over 60 years old who have online consultation experience is much smaller. Regarding the patient education level, 60.81% of the patients had a bachelor’s degree or above, and only 39.19% of the patients had below a bachelor’s degree. Regarding the frequency of patient OMC consultations, over the past year, 19.47% of the patients had frequent consultations (more than five consultations), 42.51% of the patients had less frequent consultations (more than two consultations but less than 5 consultations), and 38.02% of the patients had two or fewer consultations.

### Results of the reliability and validity tests

Composite reliability (CR) and internal consistency (Cronbach’s α) are the commonly used indicators of questionnaire reliability. The CR and Cronbach’s α values of each variable are greater than 0.6 as shown in Table 2. These results indicate that the questionnaire we developed has good reliability, following recommendation of Nunnally [27].

**Table 2:** Results of the reliability analysis

Variable	Items	Factor loading	Cronbach’s α	CR	AVE
PV	PV1	0.732	0.776	0.715	0.612
	PV2	0.686			
PT	PT1	0.783	0.821	0.783	0.636
	PT2	0.809			
IC	IC1	0.752	0.645	0.697	0.532
	IC2	0.657			

The validity testing of a questionnaire entails testing two components, namely, content validity and construct validity. Since the index expression of this study was verified by others many times, the questionnaire had high content validity. Structural validity consists of convergent and discriminant validities. Convergent validity requires the factor loading and average variance extracted (AVE) values of each index item to exceed 0.5 [28]. As shown in Table 2, all variables in this study exceeded this threshold. Discriminant validity requires the correlation coefficient of each variable to be less than the square root of the AVE value of the variable, and Table 3 reflects that the questionnaire passed the discriminant validity test.

**Table 3:** Discriminant validity matrix

Variable	PV	PT	IC
PV	0.782	N/A	N/A
PT	0.609	0.797	N/A
IC	0.276	0.518	0.729
AVE	0.612	0.636	0.532

### Results of the descriptive statistics

Table 4 lists the descriptive statistics and correlations of the main variables in the study. The results indicate that both perceived value (r = 0.827, p < 0.05) and perceived trust (r = 0.759, p < 0.05) are statistically significantly positively correlated with intention to consult. Notably, the professional title of the doctor, number of consultations, favorability rating of the doctor, and disease risk category all have an impact on patients’ intention to consult.

**Table 4:** Descriptive statistics and correlations

	Mean	SD	PV	PT	Title	Number	Rate	Risk	IC
PV	3.722	1.125	1.000						
PT	3.738	1.115	0.725**	1.000					
Title	3.159	1.067	0.361**	0.385**	1.000				
Number	3.139	0.785	0.511**	0.531**	0.605**	1.000			
Rate	3.368	0.759	0.617**	0.616**	0.311**	0.559**	1.000		
Risk	0.522	0.500	0.015	− 0.267**	− 0.062	− 0.004	− 0.072	1.000	
IC	3.964	1.387	0.827**	0.759**	0.343**	0.526**	0.671**	− 0.099*	1.000

^*^: p < 0.1; **: p < 0.05

### Results concerning the model test and moderating effect test

In this paper, the model estimation is conducted by the least-squares method. To display the relationships between the dependent variable and the independent variable and the role of the moderator variable more clearly, Table 5 shows the estimation model hierarchically. Column 1 is the model containing regression of the control variable only. Column 2 introduces the independent variable based on column 1, and column 3 introduces the interactive term on the basis of column 2. Table 5 shows that the adjusted R^2^ and F values as the variables increase are reasonable. The VIF test for each variable was less than 10, indicating that there is no multicollinearity between the variables.

**Table 5:** Parameter estimates

Independent variable	(1)	(2)	(3)
Constant	− 0.579 (− 2.070)**	− 0.775 (− 3.980)***	− 0.891 (− 3.27)***
PV		0.649 (12.100)***	0.489 (6.360)***
PT		0.308 (5.390)***	0.505 (6.410)***
Title	0.074 (1.160)	− 0.304 (− 0.680)	− 0.017 (− 0.380)
Number	0.330 (3.300)	0.073 (1.030)	0.059 (0.850)
Rate	0.995 (11.500)***	0.322 (4.620)***	0.295 (4.29)***
Risk	− 0.154 (− 1.410)	− 0.082 (− 1.000)	0.127 (0.420)
Risk*PV			0.336 (3.270)***
Risk*PT			− 0.389 (− 3.650)***
Observation	339	339	339
Adj R-squared	0.483	0.753	0.762
F	79.910	172.930	136.200

t statistics in parentheses

^**^p < 0.05; *** p < 0.01

According to column 3 of Table 5, the statistical results for perceived value (${\beta }_{1}=0.489, \mathrm{t}=6.360,\mathrm{ p}<0.01$) and perceived trust (${\beta }_{2}=0.505, \mathrm{t}=6.410,\mathrm{ p}<0.01$) are significant. Therefore, perceived value and perceived trust have a significant positive impact on the intention to consult, and H1 and H2 were confirmed. When an orthopedic patient has an online consultation, the greater his/her perceived value and perceived trust, the greater his/her intention to consult in the OMC.

**Fig. 2:**
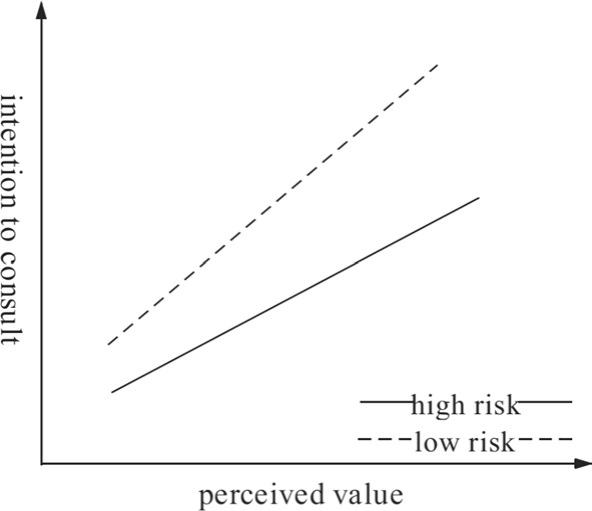
The moderating effect of disease risk category on the relationship between perceived value and intention to consult

According to column 3 of Table 5, the interaction ${R\mathrm{isk}}_{\mathrm{i}}*{PV}_{\mathrm{i}}$ (${\beta }_{7}=0.336, \mathrm{t}=3.270, \mathrm{p}<0.01$) is positive and significant. The results show that orthopedic disease risk plays a role in moderating the relationship between patients’ perceived value and their intention to consult in the OMC, and H3 was confirmed. Figure 2 illustrates that perceived value has a greater influence on the intention to consult among patients with low-risk disease than among those with high-risk disease. When patients conduct online consultations, users with low-risk diseases can describe the condition more easily, and the doctor’s diagnosis process is simpler. Low-risk diseases have a lower mortality rate and a higher cure rate. Therefore, patients with low-risk disease have a higher perceived value than patients with high-risk disease, and the greater the value the patients perceive, the greater their intention to consult in an OMC.

**Fig. 3:**
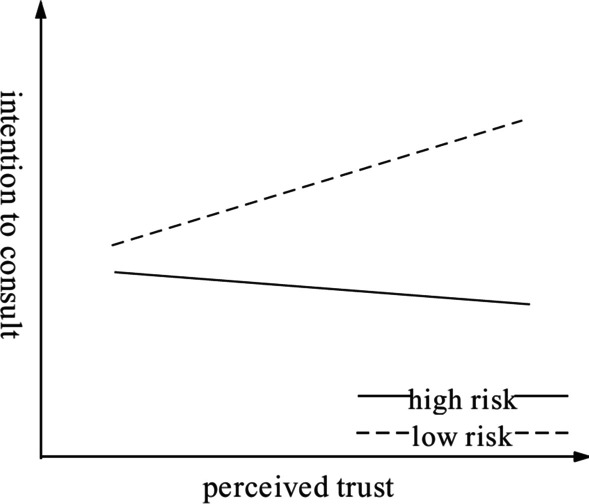
The moderating effect of disease risk category on the relationship between perceived trust and intention to consult

According to the results of the empirical model analysis, the interaction ${\mathrm{Risk}}_{\mathrm{i}}*{\mathrm{PV}}_{\mathrm{i}}$ (${\upbeta }_{8}=-0.389, \mathrm{t}=-3.650, \mathrm{p}<0.01$) is positive and significant. It is demonstrated that orthopedic disease risk category plays a role in moderating the relationship between patients’ perceived trust and their intention to consult in the OMC, and H4 was confirmed. As shown in Fig. 3, it seems that the effect of perceived trust on different categories of disease risk is the opposite, and the main effect is not significant after the interaction is offset. However, we demonstrated that perceived trust has a significant positive impact on intention to consult, indicating that the main effect of perceived trust on intention to consult is significant. In the test of the interaction term, the perceived trust of patients with low-risk disease has a positive influence on intention to consult, while the slope direction is the opposite for high-risk disease. This finding indicates that disease risk has a significant moderating effect on the relationship between perceived trust and intention to consult and that the moderating effect on patients with high-risk disease is greater than that on patients with low-risk diseases. The greater the perceived trust of low-risk disease patients, the greater their intention to seek online consultation; the perceived trust of high-risk disease patients has little effect on their intention to seek online consultation. Due to the limitations of consultations in OMC, users with high-risk diseases tend to seek medical treatment offline to receive systematic treatment as soon as possible.

### Results of the robustness analysis

To examine the robustness of the research results, disease risk category was not used as a moderator variable, but the data were divided into two subsamples according to the category of disease risk (high-risk disease and low-risk disease); regression analysis was then performed by the least-squares method. The purpose of this analysis was to verify whether perceived value and perceived trust have a significant impact on the intention to consult without the risk of disease as a moderator variable. The results of the robustness test are shown in Table 6. The results in the table are consistent with the results of the previous model with interactive item data. This finding demonstrates that perceived value and perceived trust still have significant impact on the intention to consult without disease risk as a moderator variable.

**Table 6:** Parameter estimates in the robustness analysis

Independent variable	High risk	Low risk
Constant	− 0.689 (− 3.620)***	− 0.930 (− 2.650)***
PV	0.839 (14.49)***	0.483 (5.140)***
PT	0.120 (1.970)**	0.478 (4.760)***
Title	0.020 (0.440)	− 0.071 (− 0.880)
Number	0.009 (0.11)	0.126 (1.080)
Rate	0.267 (3.400)***	0.334 (2.920)***
Observation	177	162
Adj R-squared	0.870	0.607
F	235.970	50.710

t statistics in parentheses

^*^p < 0.1; **p < 0.05; ***p < 0.01

## Discussion

This study investigated the influencing factors associated with patient intention to seek orthopedic consultations in an OMC, collected data through questionnaires and tested hypotheses by establishing empirical research models. The results of this research indicate that the perceived value and perceived trust of users have a significant positive impact on the intention to consult. The greater the patients’ perceived value and perceived trust, the greater their intention to consult in an OMC will be. This finding shows that perceived value and perceived trust are the main factors affecting whether patients choose an OMC as an electronic consultation tool.

Many orthopedic patients now choose an OMC for consultation services. The original intention of these patients was to obtain information and effective treatment advice for their condition through a convenient online consultation service. To enhance the perceived value of orthopedic patients regarding an OMC, we mainly focused on enhancing patients’ perception of functional value and emotional value. To strengthen orthopedic patients’ perception of the functional value of an OMC, it is necessary to pay attention to professional and reliable orthopedics consulting services and the pricing of consultations. Operators of an OMC need to improve the timeliness and reliability of the consulting services and the qualifications, expertise, and service attitudes of the online orthopedic surgeons. These aspects are essential for improving professional and reliable orthopedics consulting services. In addition, the pricing of online consultations is an important dimension of the patient’s perception of value, and it is necessary to promote their perception of excellent quality and a reasonable price. To enhance orthopedic patients' perception of the emotional value of online consultations, it is necessary to provide patients with a good experience of consulting services. It is significant for patients to realize that online consultation services are safe so that they can communicate with online doctors in a relaxed and comfortable manner. Online doctors need to be able to correctly understand the content (text, pictures, videos, etc. provided by patients) and be empathetic towards the patient's situation.

Because of the accuracy, privacy and sensitivity of medical information, trust is particularly important in orthopedic disease consultation. The significant positive impact of perceived trust on patients' intention to consult indicates that patients’ trust in the information and services provided by online doctors and their trust in the OMC are key factors affecting their intention to consult. To enhance the perceived trust of orthopedic patients, it is necessary to enhance their trust in orthopedic doctors in the OMC. Doctors need to protect patient privacy during confidential doctor–patient interactions and provide patients with specific suggestions for solving problems. On the other hand, it is also important to strengthen patients' trust in online platforms such that patients feel that consultations in an OMC can meet their needs and that the community’s consulting services are trustworthy. In addition to ensuring the system security of an OMC, a procedure for solving patient problems should be established. For example, when a doctor cannot provide a consultation service, the OMC should promptly recommend other doctors to guarantee that the patient has a good consultation service experience.

This study analyzed the influence of orthopedic disease category on the relationship between influencing factors and patients' intention to consult. For orthopedic patients with low-risk diseases, it is important to enhance perceived value and perceived trust. For orthopedic patients with high-risk diseases, more attention should be paid to their perception of the value of orthopedic consulting services. They could have sought offline consultations in physical hospitals, and the disease might not have been resolved (the doctor’s ability or the hospital’s surgical conditions were limited, etc.). Consultation in an OMC is another important channel for patients to obtain help. The valuable diagnosis and treatment suggestions provided by online doctors will strengthen patients’ intention to consult. In addition, orthopedic patients and doctors often have different perceptions of disease risk. An OMC can prompt doctors to judge the disease risk first when a patient consults and remind the doctors to discuss the risk with the patient.

In this study, the professional titles, number of consultations, and favorability ratings of doctors in an OMC were regarded as control variables. The favorability rating of doctors has a significant impact on patients' intention to consult. The operators of an OMC should pay full attention to the use of this evaluation mechanism. On the one hand, the system should encourage patients to give detailed feedback about their consultations, and on the other hand, doctors should be encouraged to pay attention to patient evaluations.

The present study has some limitations. First, the research scope is mainly patients in an OMC in China. However, patients’ consultation behaviors are affected by their geographical environment, living habits and thinking patterns. Future research could involve a comparative study of patients’ intention to consult in an OMC in different regions. Second, this study explores the factors influencing online orthopedic doctor–patient consultations in an OMC, but the research objects are all patients who have experience with online orthopedic doctor–patient consultations. The factors influencing online orthopedic doctor–patient consultations among patients who do not have consultation experience need to be further explored. In addition, the main research in this paper examines the influencing factors of patients’ online consultation. The factors influencing doctors’ participation in online interaction are worthy of further study.

## Conclusions

Orthopedic patients’ perceived value of and perceived trust in online consultations in an OMC could significantly influence their intention to seek online disease diagnosis and treatment consultations. The effects of perceived value and perceived trust on patients’ intention to consult significantly vary across different disease risk categories. Therefore, enhancing the perceived value and perceived trust of orthopedic patients is an important component of OMC operation and management.

In practice, the conclusion of this study has certain reference value for the operation and management of an OMC. The OMC should provide patient-centered services and enhance patients’ perceived value and perceived trust. From the perspective of the interaction and dissemination of medical information in an OMC, it is necessary to strengthen the mechanism for reviewing information in the community and avoid the dissemination of low-quality and false information, which could lead to a decline in the perceived value and perceived trust of patients. From the perspective of consulting services, it is necessary to pay attention to the entire process of patient consulting, improve the quality of consulting services, truly solve patients’ problems, and improve patients’ perceived value and perceived trust. In addition, based on the analysis of different orthopedic disease risks, the OMC should establish a response mechanism, and when the program application recognizes that a patient seeking an orthopedic consultation has a high-risk orthopedic disease, it can promptly notify the patient and doctor.

## Supplementary Information

**Additional file 1**. Questionnaire on factors influencing online orthopedic doctor-patient consultations.

## Data Availability

The datasets used and/or analyzed during the current study are available from the corresponding author on reasonable request.

## References

[1] Sims JM (2018). Communities of practice: telemedicine and online medical communities. Technol Forecast Soc Chang.

[2] Wang P, Zhou L, Mu D, Zhang D, Shao Q (2020). What makes clinical documents helpful and engaging? An empirical investigation of experience sharing in an online medical community. Int J Med Inform.

[3] Beijing Business Today. More than 30 million consultations on Good Doctor Online in 2019. 2021. http://epaper.bbtnews.com.cn/site1/bjsb/html/2020-01/07/content_442410.htm. Accessed 20 Apr 2021.

[4] Foni NO, Costa LAV, Velloso LMR, Pedrotti CHS (2020). Telemedicine: Is it a tool for orthopedics?. Curr Rev Musculoskelet Med.

[5] Cota A, Tarchala M, Parent-Harvey C, Engel V, Berry G, Reindl R, Harvey EJ (2017). Review of 5.5 years' experience using E-mail-based telemedicine to deliver orthopedic care to remote communities. Telemed E-Health..

[6] Dekker AB, Bandell D, Kortlever JTP, Inger B, Schipper IB, Ring D (2020). Factors associated with patient willingness to conduct a remote video musculoskeletal consultation. Arch Bone Joint Surg ABJS.

[7] Lambrecht CJ, Canham WD, Gattey PH, McKenzie GM (1998). Telemedicine and orthopedic care. A review of 2 years of experience. Clin Orthop Relat Res.

[8] Buvik A, Bergmo TS, Bugge E, Smaabrekke A, Wilsgaard T, Olsen JA (2019). Cost-effectiveness of telemedicine in remote orthopedic consultations: randomized controlled trial. J Med Internet Res.

[9] Thomas K, Burton D, Withrow L, Adkisson B (2004). Impact of a preoperative education program via interactive telehealth network for rural patients having total joint replacement. Orthop Nurs.

[10] Claassen A, Schers HJ, Busch V, Heesterbeek PJC, Ende CHMVD (2020). Preparing for an orthopedic consultation using an eHealth tool: a randomized controlled trial in patients with hip and knee osteoarthritis. BMC Med Inform Decis Mak.

[11] Sawhney C, Singh Y, Jain K, Sawhney R, Trikha A (2020). Trauma care and COVID-19 pandemic. J Anaesthesiol Clin Pharmacol.

[12] Miguela Álvarez SM, Bartra Ylla A, Salvador Carreño J, Castillón P, García Cardona C (2020). Telephone consultation service in orthopedics during COVID-19 pandemic. Rev Esp Cir Ortop Traumatol..

[13] Stahl I, Katsman A, Zaidman M, Keshet D, Sigal A, Eidelman M (2019). Reliability of smartphone-based instant messaging application for diagnosis, classification, and decision-making in pediatric orthopedic trauma. Pediatr Emerg Care.

[14] Daruwalla ZJ, Wong KL, Thambiah J (2014). The application of telemedicine in orthopedic surgery in Singapore: a pilot study on a secure, mobile telehealth application and messaging platform. JMIR Mhealth Uhealth.

[15] Keith K, Hansen DM, Johannessen MA (2018). Perceived value of a skills laboratory with virtual reality simulator training in arthroscopy: a survey of orthopedic surgery residents. J Am Osteopat Assoc.

[16] Choi KS, Cho WH, Lee S, Lee H, Kim C (2004). The relationships among quality, value, satisfaction and behavioral intention in health care provider choice: a South Korean study. J Bus Res.

[17] Harbishettar V, Krishna KR, Srinivasa P, Gowda M (2019). The enigma of doctor–patient relationship. Indian J Psychiatry.

[18] Audrain-Pontevia A, Menvielle L (2018). Effects of interpersonal trust among users of online health communities on patient trust in and satisfaction with their physician. Int J Technol Assess Health Care.

[19] Yang H, Guo X, Wu T (2015). Exploring the influence of the online physician service delivery process on patient satisfaction. Decis Support Syst.

[20] Venkatesh V, Davis FD (2000). A theoretical extension of the technology acceptance model: four longitudinal field studies. Manag Sci.

[21] Cengiz E, Kirkbir F (2017). Customer perceived value: the development of a multiple item scale in hospitals. Probl Perspect Manag.

[22] Ponte EB, Carvajal-Trujillo E, Escobar-Rodriguez T (2015). Influence of trust and perceived value on the intention to purchase travel online: Integrating the effects of assurance on trust antecedents. Tour Manag.

[23] Sillence E, Briggs P, Harris PR, Fishwick L (2007). How do patients evaluate and make use of online health information?. Soc Sci Med.

[24] Liu F, Li Y, Ju X (2019). Exploring patients' consultation behaviors in the online health community: the role of disease risk. Telemed J E Health.

[25] Ju C, Zhang S (2020). Influencing Factors of continuous use of web-based diagnosis and treatment by patients with diabetes: model development and data analysis. J Med Internet Res.

[26] Wang Y, Wu H, Xia C, Lu N (2020). Impact of the price of gifts from patients on physicians' service quality in online consultations: empirical study based on social exchange theory. J Med Internet Res.

[27] Nunnally JC (1978). Psychometric theory.

[28] Fornell C, Larcker DF (1981). Structural equation models with unobservable variables and measurement error: algebra and statistics. J Mark Res.

