# Supplementary material for: Factors influencing online orthopedic doctor–patient consultations
Source: BMC Med Inform Decis Mak. 2021 Dec 13;21:346. doi: 10.1186/s12911-021-01709-1 (PMC8666471; doi:10.1186/s12911-021-01709-1)
Supplement: Supplementary file 1 — Additional file 1. Questionnaire on factors influencing online orthopedic doctor-patient consultations. [file 12911_2021_1709_MOESM1_ESM.docx]

No._____________

**Questionnaire on factors influencing** **online orthopedic doctor-patient consultations**

Dear Sir/Madam：

Good days!

This is a questionnaire about factors influencing online orthopedic doctor-patient consultations (such as lumbar muscle strain, heel pain, etc.). If you have conducted online orthopedic doctor-patient consultations in online medical community(OMC), we would be grateful if you could use a few minutes to complete the following questionnaire. The research group promises that all your personal answers will only be used for academic research. Please feel free to fill in.

Your serious answer is of great significance to this study. Please fill it out truthfully. Thank you again for your cooperation!

Instructions:

1 Please do not fill in or mention your name in the questionnaire;

2 There is no right or wrong answer, just check it according to your own situation;

3 Please use "√" to check the corresponding option that matches your actual situation.

**Basic Information**

1. gender

①male ②female

2. Age

①18~30 ②31~60 ③60 and above

3 education

①senior High school and Below ②junior college

③undergraduate ④master and above

4 frequency of consultation in the OMC over the past year

①more than 5 times

②more than 2 times and less than 5 times

③less than 2 times

**Survey on factors influencing online orthopedic doctor-patient consultations**

1. disease risk category you consulting online:

①fracture of cervical vertebrae, pelvic fracture, deep venous thrombosis of lower limb, open fracture, dismemberment of limbs and trunk, etc.

②fasciitis, lumbar muscle strain, heel pain, tenosynovitis, soft tissue injury, external humeral epicondylitis, etc.

2. the professional title of the doctor you consulting online:

①resident ②attending physician ③associate chief physician ④chief physician

3. number of consultations of the doctor you consulting online:

①100 and below ②100-300 ③300-500 ④500 and above

4. favorability rating of the doctor you consulting online:

①70% and below ②70%-80% ③80%-90% ④90% and above

5. Please tick "√" in the serial number corresponding to your last online orthopedic doctor-patient consultation experience.

(1=strongly disagree; 2=disagree; 3=neither agree nor disagree; 4=agree; 5=strongly agree)

| Questions | Score | | | | |
| --- | --- | --- | --- | --- | --- |
|  | 1 | 2 | 3 | 4 | 5 |
| IC1: If I need an orthopedic treatment in the future, I will continue to use the consultation service in the online medical community. |  |  |  |  |  |
| IC2: I would recommend that other users who need orthopedic treatment seek a consultation in the online medical community. |  |  |  |  |  |
|  | | | | | |
| PV1: The online medical community can provide professional and reliable online orthopedic consulting services, and the price is reasonable. |  |  |  |  |  |
| PV2: The orthopedic consultation service provided by the online medical community is satisfying. The consultation made me feel relaxed and was enjoyable. |  |  |  |  |  |
|  | | | | | |
| PT1: The doctors in the online medical community will do their best to solve my problems and keep my consultation information confidential. |  |  |  |  |  |
| PT2: The online medical community will try its best to solve the problems during my consultation and keep my personal information confidential. |  |  |  |  |  |
|  | | | | | |

**The questionnaires are all over. Thank you again for your cooperation！**
